# Social sciences research in neglected tropical diseases 1: the ongoing neglect in the neglected tropical diseases

**DOI:** 10.1186/1478-4505-8-32

**Published:** 2010-10-21

**Authors:** Pascale Allotey, Daniel D Reidpath, Subhash Pokhrel

**Affiliations:** 1 Global Public Health, Jeffrey Cheah School of Medicine and Health Sciences, Monash University, Sunway Campus, Bandar Sunway, Malaysia; 2 Health Economics Research Group, Brunel University, West London, UK

## Abstract

Centuries of scientific advances and developments in biomedical sciences have brought us a long way to understanding and managing disease processes, by reducing them to simplified cause-effect models. For most of the infectious diseases known today, we have the methods and technology to identify the causative agent, understand the mechanism by which pathology is induced and develop the treatment (drugs, vaccines, medical or surgical procedures) to cure, manage or control.

Disease, however, occurs within a context of lives fraught with complexity. For any given infectious disease, who gets it, when, why, the duration, the severity, the outcome, the sequelae, are bound by a complex interplay of factors related as much to the individual as it is to the physical, social, cultural, political and economic environments. Furthermore each of these factors is in a dynamic state of change, evolving over time as they interact with each other. Simple solutions to infectious diseases are therefore rarely sustainable solutions. Sustainability would require the development of interdisciplinary sciences that allow us to acknowledge, understand and address these complexities as they occur, rather than rely solely on a form of science based on reducing the management of disease to simple paradigms.

In this review we examine the current global health responses to the 'neglected' tropical diseases, which have been prioritised on the basis of an acknowledgment of the complexity of the poverty-disease cycle. However research and interventions for neglected tropical diseases, largely neglect the social and ecological contextual, factors that make these diseases persist in the target populations, continuing instead to focus on the simple biomedical interventions. We highlight the gaps in the approaches and explore the potential of enhanced interdisciplinary work in the development of long term solutions to disease control.

## Introduction - The neglected tropical diseases

The Millennium Declaration provided a catalyst around which the global community, particularly world leaders, could refocus efforts and recognise responsibilities towards the most vulnerable and least served in society. It forced the world's attention onto those sections of the community left behind by the rapid progress in technology, highlighting the downward spiral of those unable to access the advantages of development.

The operationalisation of the Millennium Declaration through the Millennium Development Goals (MDGs) attempted to target the various factors that cause and sustain poverty and its consequences. These have had a significant impact on public health across a number of areas. In the area of communicable diseases there has been significant, increased activity and progress across a number of diseases that affect the poor. HIV/AIDS, malaria and tuberculosis (the "big three") are given priority in MDG 6 - collectively they account for over 5 million deaths annually [1]. Several partnerships and initiatives have been created with more 'ring fenced' resources than have ever been available to global health efforts [2]. The funds support programs, drug and vaccine developments and the scale up of various interventions. The increase in global health activity across these three diseases is reflected in increased public awareness, access to treatment, funding for research and development and ongoing monitoring and evaluation to ensure accountability and the achievement of targets.

Others have argued, however, that the focus on the big three has been at the cost of other health priorities that also adversely affect vulnerable, disenfranchised and neglected populations [3,4]. Diseases such as schistosomiasis, soil transmitted helminths, lymphatic filariasis, onchocercias, dracunculiasis, buruli ulcers, leprosy (to name a few) have a significant impact on the poor. Described as neglected tropical diseases (NTDs), these conditions fall within a category of infectious diseases that predominantly affect over a billion of the world's poorest people, are disabling, disfiguring and stigmatizing. They are 'neglected' because they are the diseases of "forgotten people" with high endemicity in rural and impoverished urban areas of low income countries and conflict and post conflict regions; the consequences are impaired childhood growth and intellectual development, low worker productivity, high maternal morbidity. These are diseases that both cause and are the result of poverty. Furthermore, there is a shortage of safe and effective treatments because these diseases present low priority markets for pharmaceutical companies [6].

Over the last five years, the WHO and the Centers for Diseases Control and Prevention (CDC) in Atlanta have identified a group of these diseases that present "targets of opportunity" based on the potential for control and possibly elimination at a low cost [7,8]. There has therefore been a significant lobby to develop a separate initiative to tackle NTDs. The lobby has had some success. Funding for NTDs has increased, as have commitments from the G8 countries and global health philanthropists. New funds from USAID for instance have been released specifically for NTD programs. Furthermore a number of pharmaceutical companies have donated products or are supporting public private partnerships, under their programs for corporate social responsibility, towards research and development for NTD control. Several global health initiative have also been established to address NTDs including: a dedicated World Health Organization department; a Global Network for NTDs, Research Partnerships and Research Initiatives and a dedicated peer reviewed academic journal. Given the current success of the NTDs in achieving the status as one of the 21^st ^century's "gang of four" [9][page e102], the issue of 'neglect', particularly by drug research and development, becomes difficult to justify.

## Current efforts to address neglected tropical diseases

The WHO NTDs program published a global plan to 2015 with details on the various strategic areas [10-12] and the web sites of various partnerships and alliances also provide updates on their activities and funded programs. Most recently, *The Lancet *journal published a series in early 2010 on NTD programs (see for instance [13-16].

Although varied the range of programs share at least one of three goals; control, elimination or eradication. The strategies for achieving these include intensified efforts on single disease vertical control programs or co-implementation across multiple disease programs where co-morbidities occur [17]. The main intervention is preventive chemotherapy delivered through mass drug administration of 'rapid impact packages [9,3].' The African Programme for Onchocerciasis Control (APOC), with donations from Merck, for instance has delivered Mectizan(tm) for onchocerciasis control since the mid 1990 s. Similar programs deliver Albendazole (donated by GlaxoSmithKline) for lymphatic filariasis. The distribution of additional preventive chemotherapies for de-worming as well as treatment of whole communities for relevant diseases in order to interrupt transmission cycles [18-21] should therefore not require major extra costs. There are also intensified efforts towards case management to identify and treat people with diseases and a renewed effort for surveillance, mapping of co-occurrence of infections and vector control.

The increase in resources for NTDs, has also seen an increase in advocacy for vaccine development described as the anti-poverty vaccine [22,23]. Hotez et al argue that vaccines against the various stages of helminth development and administered at the same time as mass drug administration would be a more sustainable intervention than existing NTD control strategies [24].

There are some broader non-disease focused plans within the global plan to combat NTDs. The promotion of intersectoral approaches under the plan is designed to enable structural change as part of an integrated package of multiple interventions. Intersectoral approaches theoretically consist of joint working across areas such as education, civil engineering and local government, not just the health sector [25]. Unfortunately, although there are good examples of where this approach has worked, it is usually implemented on a small scale, relying largely on the goodwill of individuals rather than at broader policy and implementation levels [26-28]

## The gaps

The current strategy is described as a comprehensive blueprint [19] towards "rescuing the bottom billion from poverty" [29] and in general, there has been very little criticism of the strategy and plans of various NTD programs. Programmatic indicators, such as increased distribution of drugs to vendors or distributors and increased coverage of children through schools, suggest that there is cause for optimism [14,30]. However, there are some concerns. A major problem facing the community working on controlling neglected tropical diseases is the lack of communication between the various players--researchers, policymakers, clinicians, public-private partnerships, donors, and patient advocacy groups. This lack of communication and synchronisation of efforts represents a significant missed opportunity for a comprehensive and holistic approach to sustainable outcomes. Specific gaps are discussed below.

At the technical level, there remain questions about the appropriateness of integrated chemotherapy across different age groups. There is also limited understanding of the most appropriate a) time intervals between various drug administration b) efficient delivery systems; c) monitoring and evaluation systems; d)mapping of drug interactions; e) monitoring of compliance; f) development and spread of resistance; g) parameters for movement from morbidity control to transmission containment and then elimination [31]. More urgently, significant challenges have been identified in attempts to integrate these vertical programs into often weak, horizontal health systems [32]. Attempts to coordinate national health priorities with donor reporting requirements has resulted in further fragmentation of efforts to strengthen national health system [16,33,34].

Further concerns have been raised by researchers who have either collaborated with implementation teams or worked with communities that have been targeted for integrated programs for NTD control [35,105]. Many of the concerns stem from the community responses to 'top down' approaches that expose already vulnerable communities to further subjugation. There is also often a lack of understanding by programmatic staff, of the contexts in which these programs are implemented. There is a further question, given patterns of re-emergence [36-38], about country ownership and sustainability once the attention of global health funders is turned elsewhere.

These issues relate largely to working with communities and health systems within a real life, uncontrolled environment and are raised regularly in community based programs. There have been discussions for instance on mobile versus community based distribution of treatments such as Ivermectin [39]. This study highlighted the gains in programs that involved community participation versus top down programs with expectations of community compliance. The study also emphasizes the challenges of working with community health workers who do not have a formal role in the health sector. Some of these lessons have informed APOC and other community directed interventions [40]. Other community based health programs have identified significant clashes between community and health service personnel where there was a clear lack of fit between community and health service priorities [41]. This has been particularly the case where there is a perception that programs originate from foreign countries and may not be in the best interest of communities [42]. These issues are often dismissed as quirky community based phenomena and seldom result in a significant research agenda. Where there is an associated research program, it is either focused on evaluation [42,43] or commissioned to explore the 'bottlenecks' that hinder what should otherwise be an efficacious intervention [44]. Consequently, there is no significant interdisciplinary research effort to explore solutions to NTDs that are acceptable and appropriate for communities.

There are also some more fundamental gaps that relate to understanding and addressing the more persistent social, cultural, economic and environmental factors that sustain vulnerability and high risk to these NTDs and other diseases of poverty. An understanding of the contextual factors that affect the neglected people who suffer from neglected diseases is essential to any intervention. The proposed solutions under the current strategy are public health, population based solutions, as opposed to individual clinical treatments. Therefore the strategy needs to consider the individual within their immediate social, cultural and physical environment, the structural factors that guide any choices they might have to control their health - health infrastructure, health systems, issues of access - which are in turn influenced by the political, policy and economic environment within governance structures. These are all in turn influenced by the social values that prevail within the society - equity, inclusion, human rights, regulation etc. Essentially there needs to be more than what currently is largely a tokenistic or rhetorical attempt to engage with other disciplines and sectors [12,26,44,45]. This would require an understanding of what different disciplines could contribute.

### Opportunities to address the neglected contexts of NTDs

There has been a tendency, particularly in public health research, to assume that social science research comes from a homogenous group of academic disciplines, and provides a standard set of tools (usually interviews and focus group discussions) applied and interpreted in a subjective manner. These tools then provide some information to tell us what a handful of people think or feel, which may or may not have a bearing on a pre-ordained plan for intervention. For researchers working particularly in disease focused areas, evidence that comes from social scientists that is not presented in an empirically based paradigm with which they are familiar, is dismissed as unscientific and incapable of making any real contribution [46]. The lack of understanding portrayed in this assumption would be akin to an assumption that a biomedical scientist would be just as competent in pathology or genetics as they would be in molecular biology or general practice. And further to that, an assumption that there would be similar methods that are used across all these disciplines.

"Social sciences" represent a collection of academic, theoretically grounded disciplines that share a broad interest in human society. The applied areas of various social science disciplines attempt to translate the theoretical traditions into research and evidence for practice. Anthropology for instance attempts to provide a holistic account of humans within the context of their cultures. This could include the systematic exploration of physical, biological, social, linguistic and historical artefacts that culminate to provide a picture of the area of interest. In medical anthropology, studies explore aspects of the culture that relate to health, illness and disease; from explanatory models of ill health; stigmatisation; and health practitioners usage and practice through to reasons for compliance with treatment [47-49]. Similarly, economics provides an understanding of how wealth is produced, distributed and consumed at the household level in microeconomics and at a broader societal or national level in macroeconomics. The applied area of health economics provides insights into how to address the critical issues of catastrophic expenditure, providing cost effective interventions that are affordable both for governments and for consumers, questions of financing of health systems, implications of providing good quality human resources, and tradeoffs between equity and efficiency etc. Other applied social science disciplines with an interest in health are, *inter alia*, medical sociology, social policy, political science, human geography, social epidemiology etc.

The social sciences have provided a robust evidence base and theoretical understanding for the description of the lives of neglected people [29,50-54]. Studies in anthropology and sociology provide insights into cultural perceptions and practices and how these provide meaning and frame choices for livelihoods, health and health seeking. TDR, a co-sponsored Special Program for Research and Training on Tropical Diseases, executed by WHO, has provided strong leadership in the development of research capacity and the evidence base to foster a better understanding of various social, economic and behavioural factors in tropical diseases [55-57]. There has also been a recent interest in extending research on the effects of the environment and climate change [58,59]. TDR among others, has funded research which demonstrates the effects of stigma and stigmatization [50,60-66]; and the fluidity with which gender dynamics support or hinder exposure to disease, access and ability to seek health care and the sequelae of disease [67-71]. We understand for instance that stigmatisation, social isolation and disfigurement are vulnerabilities that result from social and cultural norms of what is considered normal and who is an acceptable member of the community [51,72,73]. We also know that while there are diseases that can result in stigmatisation, stigmatisation can also result in increased exposure to disease and exacerbate the sequelae [74]. The effects of these social determinants on health are less directly observable than bacteria in a Petri-dish and therefore require interventions that are distinctly different from a drug or vaccine, operating at a socio-ecological rather than simply biological level.

The recent report from the WHO Commission on Social Determinants of Health (CSDH) also provides comprehensive data on the inequity in health outcomes for the poorer members of society [75]. The World Bank produces regular global monitoring reports on poverty and its consequences [76]. There is also some research on the effects of conflict and displacement [77-79] and the lives of the rural poor and nomadic people [80,81]. The research provides insights that are not only descriptive but also analytical in their ability to identify complex relationships between various factors. The now extensive body of work in health and development economics supports a basic understanding of household economies in families living in poverty and has also increased our understanding of poor communities' willingness and ability to pay for health related interventions [82-88]

To say the social sciences have been overlooked in global public health efforts would be inaccurate [45,89,103,104] (see also Reidpath and Pokhrel in this series). The techniques and research approaches developed through these disciplines have been packaged and applied to good effect in other areas in public health to help in the translation of biomedical research into policy and clinical and public health practice. These applications include program evaluation, health services research, health systems research and implementation research [44,82,90]. Health services research for instance develops the evidence base for financing, organization, delivery, evaluation, and outcomes of health services. Health systems research is defined to include structures, equipment, supplies, policies, people and processes [82] p0719. Implementation research is defined as applied research that aims to develop the critical evidence base that informs the effective, sustained and embedded adoption of interventions by health systems and communities. It deals with the knowledge gap between efficacy, effectiveness and current practice to produce the greatest gains in disease control [44]. There have also been recent innovations to combine environmental social determinants with epidemiological screening and surveillance tools [91]

The methods used for these applied areas include a wide suite of quantitative and qualitative research techniques driven by the specific research question and the particular conceptual framework which best captures the issue. Rigorously undertaken these all produce evidence in various forms that inform public health challenges. In spite of the potential that this research has, and the growing evidence based that is being produced, it is unclear how well this wealth of data is being applied and integrated into global health programs such as those laid out for NTD control [90,92-94].

## Conclusion

The intense advocacy that preceded the profile that NTDs currently enjoy was remarkable and indeed a systematic analysis of this process would make an important contribution to our understanding of advocacy and the dynamic research-policy-practice nexus in public health. The NTDs campaign has relentlessly highlighted the plight of the populations affected by the range of target diseases. A great deal has been made of stigmatization, disfigurement, persistent poverty, poor maternal and child health outcomes, poor health and education of children caused by infectious diseases. The choice of the word "neglect" is pointed and loaded, forcing us to reflect on our social obligations. Inherent in this campaign strategy is an appeal for the recognition of human suffering and the need for social justice [47]. However, while preventive chemotherapy and active case management might demonstrate a reduction in the incidence and prevalence of specific diseases, they do not; in and of themselves, alleviate poverty or stigmatization; improve equity or livelihoods, or change the environmental conditions which present an ongoing risk for the poor. Notwithstanding the appropriation of these broader determinants and value laden constructs in the NTD lobby process, interventions include very little to address them.

A strategy that responds to the NTDs lobby, morally and ethically requires intervention addressing the root causes of suffering and vulnerability. These causes are not however, confined to infectious diseases. Critically, the cause-effect relationship is bidirectional - poverty increases vulnerability and exposure to disease and affects access to treatment and the outcome of disease. The futility of the removal of specific diseases without addressing poverty as well has been raised time and again. More recently, the message features prominently on the home page of the WHO/CSDH and reads: "why treat people without changing what makes them sick" [95]. At the very least increasing standards of living, provision of the basic human rights of food/water, shelter, and clothing are definitive interventions towards the elimination of NTDs. The body of evidence that supports the need for structural intervention is significant [96]. Tackling structural problems is harder because the interventions required are more complex; some have suggested too complex to consider [97]. However not intervening at these levels compounds the futility of current efforts. The re-emergence of diseases that were supposed to have been eradicated 40 years ago [36] is a case in point.

The basic concern here is not new and to a significant degree, revisits the major, largely unresolved debates that raged almost 40 years ago between proponents and opponents of Primary Health Care (PHC) [98-100]. The critical question is this: does one narrowly partition out individual causes, in this instance one disease causing pathogen or another, and address them as independent context free problems amenable to context free solutions? Or alternatively is there a need for a different approach which attempts to address the multiple causes of a disease (not just the infectious agent) within the context in which they occur? The primary health care debate's answer to this question was the introduction of Selective Primary Health Care programs [101]. These vertical programs were the precursor of the current context-free, pathogen focussed NTDs strategy. Under the guise of a 'pro-poor' strategy and a vaccine against poverty, it remains a medical and technological fix; the "magical bullet" to combat disease and therefore a social problem [98].

We obviously do not underestimate the importance of biomedical technologies to managing NTDs. However it is also critical that they should not be over-estimated. Without significant efforts to address health *and *poverty, along with the myriad marginalising factors in the social, cultural, economic, political and physical environments in which affected populations live, there will continue to be neglected people and neglected contexts. Vaccines and drugs do not cure *neglect or poverty *and are not a sufficient condition (and quite possibly not even a necessary condition) to "rescue the bottom billion" from poverty [29].

To obtain the higher objective of improving health and reducing vulnerabilities, it is important for researchers, policy makers and funding agencies to broaden the perspective on the range of research and the integrated of interventions that are needed to address neglected diseases of neglected populations, and to rethink the nature of evidence to show effectiveness. There is a need to refocus on the health of neglected populations [98] - and not merely removing disease.

There are two major areas that need to be considered for a sustainable impact 'neglected' tropical diseases:

1. To extend the persistent, narrow view of health improvements based on medical interventions and technological fixes towards a broader agenda of health improvements based on dynamic process (of which medical interventions are but one of the inputs) that enable individuals to interact optimally with their environments [98].

2. To enhance the implementation of programs towards outcomes that address health as an issue that encompasses more than the absence of disease.

For this there needs to be balanced investment across areas of health research that enable an outcome that ensures the health of populations not the reduction of the incidence of a specific disease. Ultimately sustainable solutions for neglected populations will not come from eradication of infections but from the eradication of poverty [102].

## Competing interests

The authors declare that they have no competing interests.

## Authors' contributions

PA constructed first draft of the manuscript with subsequent inputs and revisions from DDR and SP. All authors read and approved the final manuscript.

## Disclaimer

The views expressed in these papers are the views of the authors and do not reflect or represent the decisions, policy or views of the World Health Organization.
